# Supplementary material for: Scedar: A scalable Python package for single-cell RNA-seq exploratory data analysis
Source: PLoS Comput Biol. 2020 Apr 27;16(4):e1007794. doi: 10.1371/journal.pcbi.1007794 (PMC7217489; doi:10.1371/journal.pcbi.1007794)
Supplement: S1 Text — Algorithm A, Algorithm B and Table A, Table B, and Table C. Includes discussion of scedar package development, minimum description length method, two-stage coding scheme for clustered scRNA-seq, mathematical theories on high-dimensional data analysis including distances between points in high dimensional space and the Johnson-Lindenstrauss lemma. Discussion of the skewed root division of a hierarchical agglomerative clustering tree. (DOCX) [file pcbi.1007794.s001.docx]

Supplementary materials for Scedar: a scalable Python package for single-cell RNA-seq exploratory data analysis

Yuanchao Zhang^1,2^, Man S. Kim^1^, Erin R. Reichenberger^1^, Ben Stear^1^, and Deanne M. Taylor^1,3^
1. Department of Biomedical and Health Informatics, The Children’s Hospital of Philadelphia, Philadelphia, PA 19041, USA

2. Department of Genetics, Rutgers University, Piscataway, NJ 08854, USA

3. Department of Pediatrics, Perelman School of Medicine, University of Pennsylvania, Philadelphia, Pennsylvania, 19104, USA

# Supplementary methods

## scedar package development

Scedar is built upon various high-performance scientific computing and visualization packages. Scedar is also extensively benchmarked and tested by unit testing, with comprehensive coverage on statements and branches.

At the time of publication, scedar uses the following packages and versions:

- numpy version 1.18.1 [[1]](https://paperpile.com/c/YTPw0C/AO52L) for matrix representation and operations.
- scipy version 1.4.1 [[2]](https://paperpile.com/c/YTPw0C/bQ9Yp) for fast Gaussian kernel density estimation, hierarchical clustering and sparse matrix.
- matplotlib version 3.1.0 [[3]](https://paperpile.com/c/YTPw0C/pfwB0) and seaborn [[4]](https://paperpile.com/c/YTPw0C/W4jdg) for visualization.
- pandas version 0.25.3 [[5]](https://paperpile.com/c/YTPw0C/1wzvf) for data frame representation.
- scikit-learn version 0.21.0 [[6]](https://paperpile.com/c/YTPw0C/t3jW8) for parallel computation of pairwise distances, k-nearest neighbor (KNN) data structure, PCA and t-SNE.
- XGBoost version 0.90 [[7]](https://paperpile.com/c/YTPw0C/uSJuQ) for scalable gradient boosting tree.
- networkx version 2.4 [[8]](https://paperpile.com/c/YTPw0C/QlSyE) for graph data structure and visualization.
- ForceAtlas2 version 0.3.5 [[9]](https://paperpile.com/c/YTPw0C/08Frv) for scalable force-directed graph layout.
- nmslib version 2.0.5 [[10]](https://paperpile.com/c/YTPw0C/gKe8) for approximate nearest neighbor search using Hierarchical Navigable Small World Graphs.
- Leidenalg version 0.7 [[11]](https://paperpile.com/c/YTPw0C/utJH) for community detection.
- umap-learn version 0.3.10 [[12,13]](https://paperpile.com/c/YTPw0C/24qD+dKPQ) for computing UMAP embeddings.
- python-igraph version 0.7.1.post6 [[14]](https://paperpile.com/c/YTPw0C/aWKB) for constructing KNN graph data structure.

We use the Python package pytest version 5.2.0 [[15]](https://paperpile.com/c/YTPw0C/gCpF) as the unit testing framework to ensure that scedar has expected behaviors, even after major coding changes. We tested each member of the package with multiple testing environments, in order to make sure that all statements and branches are executed in the tests, i.e. comprehensive code coverage. The code coverage was measured by the Python package coverage (v 5.03, https://coverage.readthedocs.io/en/coverage-5.0.3/). Although comprehensive testing coverage does not guarantee that the package is bug-free, it eliminates obvious errors, e.g. accessing local variables before definition.

Comprehensive unit testing greatly helps with validating correctness, ensuring reproducibility and refactoring the code. We carefully tested our analytical procedures with multiple input datasets to cover standard and edge cases, in order to make sure that the results are correct and reproducible. We also confidently refactored the code multiple times throughout the development process to improve backend performance, accommodate special use cases, and reorganize intra-package dependencies. For a non-trivial package with multiple interrelated components like scedar, changes in certain components may unexpectedly affect other components that directly or indirectly use the changed ones, so that validating the correctness after the changes would require a significant amount of effort without the comprehensive tests we deliberately built into scedar.

## Minimum description length

Minimum description length (MDL) is the minimum information size required to describe a set of data by a model [[16]](https://paperpile.com/c/YTPw0C/JtKHw). If the model plainly describes the data verbatim, the MDL of the data is equivalent to the size of the data. The MDL of the data can be reduced by a more sophisticated model that exploits the statistical properties in the data. For example, if 95% of the entries in a 10,000 x 10,000 matrix are 0s, a model can record only the indices of non-0 entries and keep a note that all other entries are 0s, which would be able to greatly reduce the MDL of the matrix. However, we do not want the model to become so complex that the information size for describing the model is very large. For example, a sequence of 10 single digit decimal integers can be described by a n=1-100 index model that stores all possible sequences, which is clearly larger than plainly describing the original 10 integers, and thus would not be a good model in the MDL framework. The principle of MDL is applied in statistics and machine learning to select the model that requires the smallest size of information to describe the model and data, and the practice is reviewed by Hansen and Yu in detail [[16]](https://paperpile.com/c/YTPw0C/JtKHw).

Using the principle of MDL, we developed a single-cell clustering method for scRNA-seq data, MIRAC. In the context of single-cell analysis, MIRAC finds the partition of cells (columns) in the matrix that yields the shortest code length of the data. The input data of MIRAC from scRNA-seq data could be any $n\times m$ matrix $X$ with $n$ cells (columns) and $m$ features (rows), where the features could be any measurements containing information about the similarity between the cells, e.g. the number of reads mapped to certain genes, distances to certain cells from a common coordinate, or dimensionality reduced coordinates in a feature space. In order to code the $n\times m$ data matrix $X$, we use a two-stage scheme [[16]](https://paperpile.com/c/YTPw0C/JtKHw), in which we code the partition of cells in the first stage and the partitioned data in the second, which we discuss in the following sections.

### Minimum description length in practice

Practically, let an observation $x$ of a random variable $X$ follow an arbitrary probability distribution $P$ with parameters $\Theta=\{\theta_{1}, \theta_{2},\ldots,\theta_{k}\}$. If $P$ is continuous, let $f$ be its probability density function, otherwise probability mass function. Then, the code length of $x$ is $-\log f(x)$ with an arbitrary base of $2$ or the Euler's number $e$. In scedar, we consistently use $e$ as the base. The code length of $n$ observations $\{x_{1}, x_{2}, \ldots, x_{n}\}$ is the sum of the code lengths of individual observations, which gives an overall code length of $\sum_{i=1}^{n} -\log f(x_{i})$. The code length of $P$ is the code length of $\Theta$ using uniform distribution for each parameter.

In a two-stage coding scheme, the overall code length of the data, i.e. the observations, is the sum of the following:

- Stage 1: code length of the statistical model $\mathcal{M=\{}P, \Theta\}$ (for the observations).
- Stage 2: code length of the observations encoded using $\mathcal{M}$.

When there are multiple statistical models ${\mathcal{\{M}}_{1},\mathcal{M}_{2},\ldots,\mathcal{M}_{t}\}$, we select the one that gives the shortest overall code length of the data. Intuitively, the closer the assumed distribution $P$ to the true distribution of the observations, the shorter the overall code length of the observations. The simpler the assumed distribution $P$, the shorter the code length of $P$.

Importantly, the code length of observations encoded by a discrete model cannot be directly compared to the code length of the same observations encoded by a continuous model. For example, let $X=[1, 1, 0, 1, 0]$ be our observations. The code length of $X$ encoded by $Bernoulli(0.6)$ is $L\left( X, Bernoulli\left( 0.6 \right) \right)=-3\log0.6-2\log0.4\approx3.365$. The code length of $X$ encoded by $Uniform(0, 1)$ is $L\left( X, Uniform\left( 0, 1 \right) \right)=-3\log1-2\log1=0$. Although $Bernoulli(0.6)$ better describes $X$ than$Uniform(0, 1)$, $L\left( X, Bernoulli\left( 0.6 \right) \right)>L\left( X, Uniform\left( 0, 1 \right) \right)$.

The theoretical background of MDL is extensively reviewed by Hansen and Yu [[16]](https://paperpile.com/c/YTPw0C/JtKHw).

### Two-stage coding scheme for clustered scRNA-seq data

In order to clarify the coding scheme for scRNA-seq clustering analysis, we introduce the following definitions and notations:

- Denote an *ordered list* $Z$ of $n$ items as $\left\langle z_{1},z_{2},\ldots,z_{n} \right\rangle$.
- Define function $list\left( \left\{ s_{1},s_{2},\ldots,s_{n} \right\} \right)=\left\langle s_{1},s_{2},\ldots,s_{n} \right\rangle$ to convert a set to a list.
- Let an $n\times m$ matrix $X$ be the data matrix of $n$ cells and $m$ features. We define the following operations:
  - $X_{i, \cdot}$ gives the $i$th row of $X$.
  - $X_{\cdot, j}$ gives the $j$th column of $X$.
  - $X_{i, j}$ gives the entry of $X$ at $i$th row and $j$th column.
  - $X_{\left\langle i_{1},i_{2},\ldots, i_{r} \right\rangle, \left\langle j_{1},j_{2},\ldots, j_{c} \right\rangle}$ gives a matrix of crossed entries of $\left\langle i_{1},i_{2},\ldots, i_{r} \right\rangle$ rows and $\left\langle j_{1},j_{2},\ldots, j_{c} \right\rangle$ columns in $X$.
- Define a *partition* $P$ of a set $S$ as a set of non-empty subsets of $S$ that are disjoint, of which the union is the same as $S$. For example, $\left\{ \left\{ 1 \right\}, \left\{ 2, 3 \right\} \right\}$ is a partition of $\left\{ 1, 2, 3 \right\}$, whereas $\left\{ \left\{ 1 \right\}, \left\{ 2 \right\} \right\}$ or $\left\{ \{\}, \left\{ 1,2,3 \right\} \right\}$ is not a partition of $\left\{ 1, 2, 3 \right\}$.
- Define operation $\left| S \right|$ on any set $S$ to give the number of elements in $S$, i.e. cardinality of $S$.
- We use a partition of a set of $n$ different integers to denote a possible clustering result of $n$ cells.
- For any partition $P=\left\{ P_{1},P_{2},\ldots,P_{k} \right\}$ of $S$:
  - $P$ is a *singleton partition* if $k=1$.
  - We call $P_{i}$ the $i$th *cluster*, where $i\in\left\{ 1, 2, \ldots, k \right\}$.
  - We define function $I(i, P)$ on any element $i\in S$, and $I\left( i, P \right)=j$ such that $i\in P_{j}$. Thus, we have a pair $\left( i, I\left( i, P \right) \right)$ for each element $i\in S$, and we call $I(i, P)$ as the *cluster label* of $i$.
  - Let $B$ be the list of cluster labels $\left\langle I\left( 1, P \right), I\left( 2, P \right), \ldots, I(n, P) \right\rangle$.

For any $X_{n\times m}$, $P=\left\{ P_{1},P_{2},\ldots, P_{k} \right\}$, and cluster labels $B$, we encode $X$ in the following two stages:

- Encode $B$ using categorical distribution. The code length of $B$ is

$$L\left( B \right)= \sum_{i=1}^{k} -\left| P_{i} \right|\log\frac{\left| P_{i} \right|}{n},$$

- Encode $X$ as $\{X_{\mathrm{list}\left\{ P_{1} \right\}, \cdot}, X_{\mathrm{list}\left\{ P_{2} \right\}, \cdot},\ldots, X_{\mathrm{list}\left\{ P_{k} \right\}, \cdot}\}$. Within each row subset $X_{\mathrm{list}\left\{ P_{z} \right\}, \cdot}$ of $X$, $m$ features are coded as individual random variables following arbitrary distributions. The code length of $X_{\mathrm{list}\left\{ P_{z} \right\}, \cdot}$ is

$$L\left( X_{\mathrm{list}\left\{ P_{z} \right\}, \cdot} \right)=\sum_{j=1}^{m} \sum_{i\in P_{z}} -\log f_{j}(X_{i,j}),$$

where $f_{j}$ is the probability density or mass function of the assumed distribution of $X_{\mathrm{list}\left( P_{z} \right),j}$.

We write the code length of $X$ with partition $P$ as $L(X, P)$. When $P$ is a singleton partition, we omit $P$ and write $L(X)$.

## Mathematical theories on high-dimensional data analysis

The following two mathematical results on high-dimensional data analysis guided our development of analytical methods for scRNA-seq datasets.

### Distances between points in high-dimensional space

As the number of features increases, all samples become closer in similarity metrics to each other [[17]](https://paperpile.com/c/YTPw0C/Krw1a), in a sense that that the distance between a sample and its nearest sample approaches to the distance between the sample and its farthest sample [[18,19]](https://paperpile.com/c/YTPw0C/g1XHq+fmwNA). This property of distance in high-dimensional space is also called distance concentration effect [[20]](https://paperpile.com/c/YTPw0C/n3vy4). Therefore, analytical methods based on distances, such as hierarchical agglomerative clustering, are less stable or, in other words, more susceptible to noise in the data. This result is mathematically described in the context of the nearest neighbors of a query point as following.

Definitions:

- Let any positive integer $m$ be the variable that the distance distributions may converge under. The variable $m$ can be interpreted as dimensionality, but this interpretation is not required by the proof of **Theorem 1** given by Beyer *et al*. [[18]](https://paperpile.com/c/YTPw0C/g1XHq).
- Let $n$ be the number of points.
- Let $X_{1}^{m}, X_{2}^{m}, \ldots, X_{n}^{m}$ be $n$ independent points such that $X_{i}^{m} \sim P_{X}^{m}$ for any $i\in\left\{ 1, 2, \ldots, n \right\}$, where $P_{X}^{m}$ a probability distribution.
- Let $Q^{m}$ be a query point sampled from the probability distribution $P_{Q}^{m}$ independently from $X_{1}^{m}, X_{2}^{m}, \ldots, X_{n}^{m}$.
- Let $0<p<\infty$ be a constant.
- Define $D_{m}(X_{i}^{m}, Q^{m})$ for any $i\in\left\{ 1, 2, \ldots, n \right\}$ as a function that returns a non-negative real number.
- Denote $D_{m}^{\min}=\min\left\{ D_{m}\left( X_{i}^{m}, Q^{m} \right) | i\in\left\{ 1, 2, \ldots, n \right\} \right\}$.
- Denote $D_{m}^{\max}= \max\left\{ D_{m}\left( X_{i}^{m}, Q^{m} \right) | i\in\left\{ 1, 2, \ldots, n \right\} \right\}$.

**Theorem 1.** *(Beyer et al.)* *If*

$$\lim_{m\to\infty} \mathrm{var}(\frac{\left( D_{m}\left( X_{1}^{m}, Q^{m} \right) \right)^{p}}{\mathbf{E}\left[ \left( D_{m}\left( X_{1}^{m}, Q^{m} \right) \right)^{p} \right]})=0,$$

*then for every* $\epsilon>0$

$$\lim_{m\to\infty} P\left[ D_{m}^{\max}\leq\left( 1+\epsilon\right)D_{m}^{\min} \right]=1.$$

The proof of **Theorem 1** is given by Beyer *et al.* [[18]](https://paperpile.com/c/YTPw0C/g1XHq). From the theorem, given that the distance distribution follows certain condition as $m$ increases, the distances of all points to the query point converges to a constant, which implies that the concept of nearest neighbor may not be meaningful [[18,19]](https://paperpile.com/c/YTPw0C/g1XHq+fmwNA). The extent of restrictiveness of the precondition is also discussed by Beyer *et al*. [[18]](https://paperpile.com/c/YTPw0C/g1XHq).

Although this property of distance between high-dimensional points affects analytical methods relying on distances [[18,19]](https://paperpile.com/c/YTPw0C/g1XHq+fmwNA), the influences could be alleviated by dimensionality reduction, of which the performance in preserving the pairwise distances is characterized by the Johnson–Lindenstrauss lemma (**Theorem 2**) [[21]](https://paperpile.com/c/YTPw0C/CNn7V).

### Johnson–Lindenstrauss lemma

The Johnson–Lindenstrauss lemma generally states that $n$ high-dimensional points in Euclidean space can be embedded into a lower dimensional Euclidean space with $O\left( \log n/\epsilon^{2} \right)$ dimensions for any $0<\epsilon<1$, while preserving the pairwise distances between $n$ points with errors within a factor of $\epsilon$ [[21,22]](https://paperpile.com/c/YTPw0C/7xpuX+CNn7V). The mathematical description of the theorem is summarized by Dasgupta and Gupta [[22]](https://paperpile.com/c/YTPw0C/7xpuX) as the following:

**Theorem 2.** *(Johnson–Lindenstrauss lemma) For any* $0<\epsilon<1$ *and any integer* $n$*, let* $k$ *be a positive integer such that*

$$k\geq4\left( \frac{\epsilon^{2}}{2}-\frac{\epsilon^{3}}{3} \right)^{-1}\ln n .$$

*Then for any set* $V$ *of* $n$ *points in* $\mathbb{R}^{d}$*, there is a map* $f:\mathbb{R}^{d}\to\mathbb{R}^{k}$ *such that for all* $u, v\in V$*,*

$$\left( 1-\epsilon\right)u-v^{2}\leq f\left( u \right)-f\left( v \right)^{2}\leq\left( 1+\epsilon\right)u-v^{2} .$$

*Further this map can be found in randomized polynomial time.*

The proof of the Johnson–Lindenstrauss lemma with elementary probabilistic techniques is given by Dasgupta and Gupta [[22]](https://paperpile.com/c/YTPw0C/7xpuX).

## Skewed root division of a hierarchical agglomerative clustering tree

We implemented a simple procedure to skew a hierarchical agglomerative clustering (HAC) before dividing the root into left and right subtrees (**Fig** **S10**). The skewed tree ensures that the smaller subtree of the root has $\geq n_{\min}^{\mathrm{cluster}}$ leaves, while preserves the ordering of leaves and maintains the invariants of a HAC tree. When $n_{\min}^{\mathrm{cluster}}$ is equal to half of the number of all leaves, the resulting division is similar to a balanced one.

Skewed root division is optional in Minimum description length (MDL) iteratively regulated agglomerative clustering (MIRAC). The procedure could be used to ensure that the divided sub-clusters are not too small, in order to improve the robustness of MDL estimation, because MDL estimation of a sub-cluster of too few samples is susceptible to noise.

**Algorithm A**: Impute gene dropouts by k-nearest neighbors

**Input**:

$X$, a numeric data matrix of size $n\times m$

$S$, a numeric k-nearest neighbor matrix of size $n\times n$

$x^{\text{min}}$, minimum of an entry in $X$ to be considered as transcribed

$k$, the number of nearest neighbors to check for imputing gene dropouts

$n^{\text{dropout}}$($\leq k$), for a zero entry $X\left[ i, j \right]$ to be called a dropout, the minimum number of cells in $k$ nearest neighbors of cell $i$ transcribing the gene $j$

$n^{\text{iter}}$, the number of iterations

**Output**:

$A$, a numeric matrix of size $n\times m$ storing the imputed transcription levels

1. $A$ ← zeros(n,m); E ← $X\geq x_{\text{min}}$ // $E\left[ i, j \right]$ stores whether gene $j$ is transcribed in cell $i$

2   **for** $i$ ← 1 **to** $n^{\text{iter}}$ **by** 1 **do**

3   $n_{i}^{\text{dropout}}$ ← $n^{\text{dropout}}$ + $\left\lceil\frac{n^{\text{iter}}-i}{n^{\text{iter}}}(k-n^{\text{dropout}}) \right\rceil$ // dropout threshold at $i$th iteration

4   **for** $j$ ← 1 **to** $n$ **by** 1 **do**

5   $I_{j\text{knn}}$ ← kNNIndices($S$, $j$) // find k-nearest neighbor indices of $j$th cell

6   **for** $g$ ← 1 **to** $m$ **by** 1 **do** // iterate all genes to impute dropout

7   **if** $X[j, g] = 0$ **then** // potential dropout

8   $n_{j\text{knn}}^{\text{exp}}$ ← sum($E[I_{j\text{knn}}, g]$)

9   **if** $n_{j\text{knn}}^{\text{exp}}$ ≥ $n_{i}^{\text{dropout}}$ **then** // impute the gene dropout

10   $A[j, g]$ ← median($X[ I_{j\text{knn}}, g]$)

11   $X[j, g]$ ← $A[j, g]$

12   $E[j,g]$ ← $A[j,g] \geq x_{\text{min}}$

13   **end**

14   **end**

15   **end**

16   **end**

17  **end**

18 **return** $A$

**Algorithm B**: Detect rare transcriptomic profiles by k-nearest neighbors

**Input**:

$S$, a numeric k-nearest neighbor matrix of size $n\times n$

$s^{\text{sim}}$ (scalar), the minimum similarity of between two cells called similar

$k$, the minimum number of nearest neighbors that are similar to a non-rare cell

$n^{\text{iter}}$, the number of iterations

**Output**:

$A$, an indicator vector of length $n$ storing whether a cell is rare or not

1   $A$ ← zeros($n$)

2 $s^{\text{min}}$ ← min($S$) // $s^{\text{min}}$ is a scalar of the overall minimum of $S$

3 $s^{\text{max}}$ ← max($S$) // $s^{\text{max}}$ is a scalar of the overall maximum of $S$

4 **if** $s^{\text{sim}}$ ≤ $s^{\text{min}}$ **then** **return** $A$ // all cells are similar

5 **else if** $s^{\text{sim}}$ > $s^{\text{max}}$ **then** **return** $A + 1$ // all cells are not similar

6   **for** $i$ ← 1 **to** $n^{\text{iter}}$ **by** 1 **do**

7   $s_{i}^{\text{sim}}$ ← $s^{\text{sim}}$ + $\left\lceil\frac{n^{\text{iter}}-i}{n^{\text{iter}}}(s^{\text{sim}}-s^{\text{min}}) \right\rceil$ // min. similarity threshold at $i$th iter.

8   **for** $j$ ← 1 **to** $n$ **by** 1 **do**

9   **if** $A[j]$ ̸= 1 **then** // cell j is not marked as rare yet

10  $s_{j,k}$ **←** kthNNSimilarity($S$, $j$, $k$, $A$) // use $A$ to ignore rare cells

11  **if** $s_{j,k}$ < $s_{i}^{\text{sim}}$ **then** $A[j] = 1$ // mark cell $j$ as rare

12   **end**

13   **end**

14  **end**

15 **return** $A$

**Table A.** Number of cell clusters for benchmark


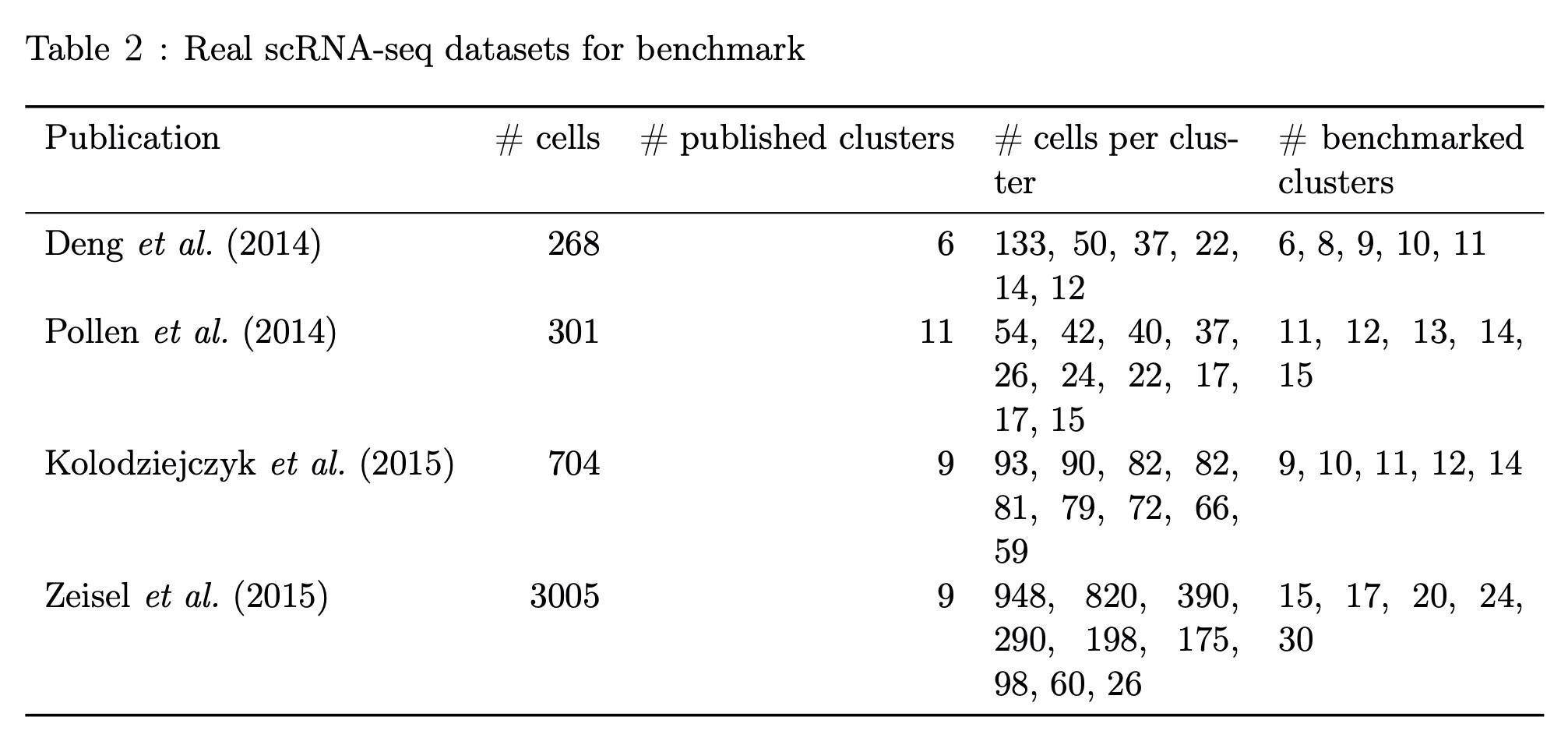


**Table B.** Top 20 Important Cluster ⟨1, 15, 22⟩ Separating Genes


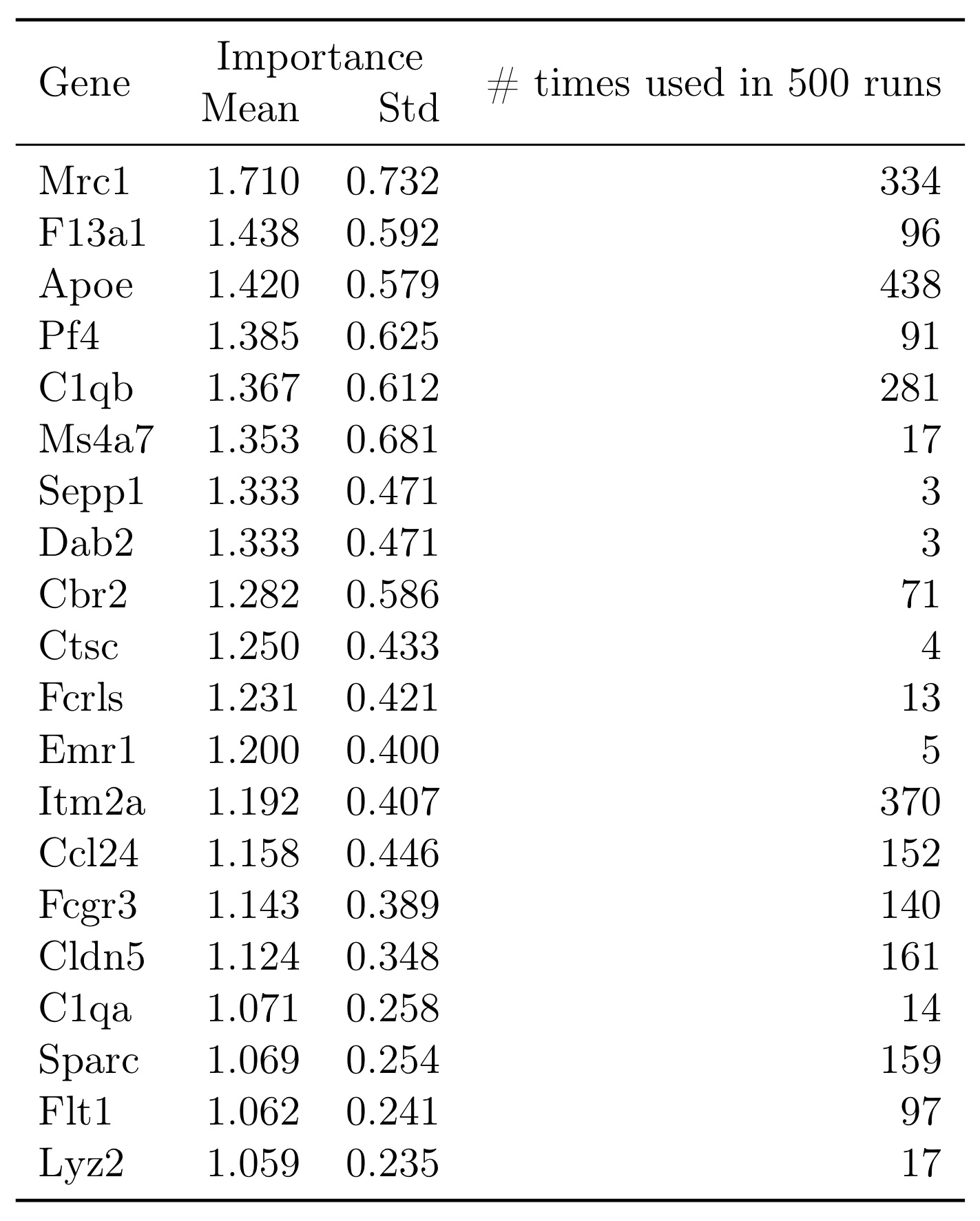


**Table C.** Software testing coverages of scRNA-seq data analysis tools. The tools are selected from the list curated by Zappia et al. [[32]](https://paperpile.com/c/YTPw0C/DAsA).

| Package | Language | Package management | Test coverage | |
| --- | --- | --- | --- | --- |
|  |  |  | Qualitative | Quantitative |
| Scedar | Python | pip | comprehensive | 100% |
| slingshot | R | bioconductor | extensive | 87% |
| ZINB-WaVE | R | bioconductor | extensive | 81% |
| Scater | R | bioconductor | extensive | 69% |
| Splatter | R | bioconductor | extensive | 69% |
| BASiCS | R/C++ | bioconductor | extensive | 61% |
| TraCeR | Python | pip/conda | extensive | NA |
| Seurat | R/C++/Java | CRAN | extensive | NA |
| MAST | R | bioconductor | extensive | NA |
| Monocle | R/C++ | bioconductor | extensive | NA |
| PHATE | Python/R/Matlab | pip/cran/source | extensive | NA |
| Scanpy | python | pip | extensive | NA |
| pySCENIC | Python | pip | extensive | NA |
| scphaser | R | CRAN | extensive | NA |
| scran | R | source | extensive | NA |
| ZIFA | Python | source | extensive | NA |
| MAGIC | Python/R/Matlab | pip/cran/source | limited | NA |
| SCDE | R/c++/fortran | source | limited | NA |
| scLVM | Python/R | pip | limited | NA |
| scPipe | R | bioconductor | limited | NA |
| scruff | R | devtools source | limited | NA |
| SC3 | R/C++ | bioconductor | none | 0 |
| BackSPIN | Python | pip/conda | none | 0 |
| Mpath | R | source tarball | none | 0 |
| SAUCIE | Python | source | none | 0 |
| SAVER | R | bioconductor | none | 0 |
| scDD | R | bioconductor | none | 0 |
| GiniClust2 | R | source | none | 0 |
| SCnorm | R | bioconductor | none | 0 |
| scMCA | R | source | none | 0 |
| scmap | R | source | none | 0 |
| SCODE | R | source | none | 0 |
| SCope | JavaScript/Python | npm | none | 0 |
| SCENIC | R | source | none | 0 |
| scImpute | R | source | none | 0 |
| scploid | R | source | none | 0 |
| Scrublet | Python | pip | none | 0 |
| scRutiNy | Python | pip | none | 0 |
| scTCRseq | Python | source | none | 0 |
| scTDA | Python | pip | none | 0 |
| SCUBA | Matlab | source | none | 0 |
| scVAE | Python | source | none | 0 |
| SIMLR | R | bioconductor | none | 0 |
| SINCERA | R | devtools source | none | 0 |
| SinQC | Python/R | source | none | 0 |
| SLICER | R | CRAN | none | 0 |
| SLICE | R | devtools source | none | 0 |
| SPADE | R | devtools source | none | 0 |
| StemID | R | devtools source | none | 0 |
| STEMNET | R | devtools source | none | 0 |
| URD | R | devtools source | none | 0 |
| Wishbone | Python | source | none | 0 |

# References

1. [Oliphant TE. A guide to NumPy. Trelgol Publishing USA; 2006.](http://paperpile.com/b/YTPw0C/AO52L)

2. [Virtanen P, Gommers R, Burovski E, Oliphant TE, Cournapeau D, Weckesser W, et al. scipy/scipy: Scipy 1.1.0rc1. 2018. doi:](http://paperpile.com/b/YTPw0C/bQ9Yp)[10.5281/zenodo.1218715](http://dx.doi.org/10.5281/zenodo.1218715)

3. [Hunter JD. Matplotlib: A 2D Graphics Environment. Comput Sci Eng. 2007;9: 90–95.](http://paperpile.com/b/YTPw0C/pfwB0)

4. [Waskom M, Botvinnik O, O’Kane D, Hobson P, Lukauskas S, Gemperline DC, et al. mwaskom/seaborn: v0.8.1 (September 2017). 2017. doi:](http://paperpile.com/b/YTPw0C/W4jdg)[10.5281/zenodo.883859](http://dx.doi.org/10.5281/zenodo.883859)

5. [McKinney W, Others. Data structures for statistical computing in python. Proceedings of the 9th Python in Science Conference. Austin, TX; 2010. pp. 51–56.](http://paperpile.com/b/YTPw0C/1wzvf)

6. [Pedregosa F, Varoquaux G, Gramfort A, Michel V, Thirion B, Grisel O, et al. Scikit-learn: Machine Learning in Python. J Mach Learn Res. 2011;12: 2825–2830.](http://paperpile.com/b/YTPw0C/t3jW8)

7. [Chen T, Guestrin C. XGBoost: A Scalable Tree Boosting System. Proceedings of the 22nd ACM SIGKDD International Conference on Knowledge Discovery and Data Mining - KDD ’16. New York, New York, USA: ACM Press; 2016. pp. 785–794.](http://paperpile.com/b/YTPw0C/uSJuQ)

8. [Hagberg A, Swart P, S Chult D. Exploring network structure, dynamics, and function using NetworkX. Los Alamos National Lab.(LANL), Los Alamos, NM (United States); 2008. Available:](http://paperpile.com/b/YTPw0C/QlSyE) <https://www.osti.gov/biblio/960616>

9. [Jacomy M, Venturini T, Heymann S, Bastian M. ForceAtlas2, a continuous graph layout algorithm for handy network visualization designed for the Gephi software. PLoS One. 2014;9: e98679.](http://paperpile.com/b/YTPw0C/08Frv)

10. [Malkov YA, Yashunin DA. Efficient and robust approximate nearest neighbor search using Hierarchical Navigable Small World graphs. arXiv [cs.DS]. 2016. Available:](http://paperpile.com/b/YTPw0C/gKe8) <http://arxiv.org/abs/1603.09320>

11. [Traag VA, Waltman L, van Eck NJ. From Louvain to Leiden: guaranteeing well-connected communities. Sci Rep. 2019;9: 5233.](http://paperpile.com/b/YTPw0C/utJH)

12. [McInnes L, Healy J, Melville J. UMAP: Uniform Manifold Approximation and Projection for Dimension Reduction. arXiv [stat.ML]. 2018. Available:](http://paperpile.com/b/YTPw0C/24qD) <http://arxiv.org/abs/1802.03426>

13. [McInnes L, Healy J, Saul N, Großberger L. UMAP: Uniform Manifold Approximation and Projection. Journal of Open Source Software. 2018;3: 861.](http://paperpile.com/b/YTPw0C/dKPQ)

14. [Csardi G, Nepusz T. The igraph software package for complex network research. InterJournal. 2006. p. 1695. Available:](http://paperpile.com/b/YTPw0C/aWKB) <http://igraph.org>

15. [Krekel H, Oliveira B, Pfannschmidt R, Bruynooghe F, Laugher B, Bruhin F. pytest 5.2.0. 2004. Available:](http://paperpile.com/b/YTPw0C/gCpF) <https://github.com/pytest-dev/pytest>

16. [Hansen MH, Yu B. Model Selection and the Principle of Minimum Description Length. J Am Stat Assoc. 2001;96: 746–774.](http://paperpile.com/b/YTPw0C/JtKHw)

17. [Domingos P. A Few Useful Things to Know About Machine Learning. Commun ACM. 2012;55: 78–87.](http://paperpile.com/b/YTPw0C/Krw1a)

18. [Beyer K, Goldstein J, Ramakrishnan R, Shaft U. When Is “Nearest Neighbor” Meaningful? Database Theory — ICDT’99. Springer Berlin Heidelberg; 1999. pp. 217–235.](http://paperpile.com/b/YTPw0C/g1XHq)

19. [Aggarwal CC, Hinneburg A, Keim DA. On the Surprising Behavior of Distance Metrics in High Dimensional Space. Database Theory — ICDT 2001. Springer Berlin Heidelberg; 2001. pp. 420–434.](http://paperpile.com/b/YTPw0C/fmwNA)

20. [Zimek A, Schubert E, Kriegel H-P. A survey on unsupervised outlier detection in high-dimensional numerical data. Stat Anal Data Min. 2012;5: 363–387.](http://paperpile.com/b/YTPw0C/n3vy4)

21. [Johnson WB, Lindenstrauss J. Extensions of Lipschitz mappings into a Hilbert space. Contemp Math. 1984;26: 1.](http://paperpile.com/b/YTPw0C/CNn7V)

22. [Dasgupta S, Gupta A. An elementary proof of the Johnson-Lindenstrauss lemma. International Computer Science Institute, Technical Report. 1999; 99–006.](http://paperpile.com/b/YTPw0C/7xpuX)

23. [Hubert L, Arabie P. Comparing partitions. J Classification. 1985;2: 193–218.](http://paperpile.com/b/YTPw0C/2ryD)

24. [Macosko EZ, Basu A, Satija R, Nemesh J, Shekhar K, Goldman M, et al. Highly Parallel Genome-wide Expression Profiling of Individual Cells Using Nanoliter Droplets. Cell. 2015;161: 1202–1214.](http://paperpile.com/b/YTPw0C/HBmnX)

25. [Zheng GXY, Terry JM, Belgrader P, Ryvkin P, Bent ZW, Wilson R, et al. Massively parallel digital transcriptional profiling of single cells. Nat Commun. 2017;8: 14049.](http://paperpile.com/b/YTPw0C/2br3J)

26. [Han X, Wang R, Zhou Y, Fei L, Sun H, Lai S, et al. Mapping the Mouse Cell Atlas by Microwell-Seq. Cell. 2018;172: 1091–1107.e17.](http://paperpile.com/b/YTPw0C/ceMr)

27. [Cao J, Spielmann M, Qiu X, Huang X, Ibrahim DM, Hill AJ, et al. The single-cell transcriptional landscape of mammalian organogenesis. Nature. 2019; 1.](http://paperpile.com/b/YTPw0C/HGk4)

28. [Pollen AA, Nowakowski TJ, Shuga J, Wang X, Leyrat AA, Lui JH, et al. Low-coverage single-cell mRNA sequencing reveals cellular heterogeneity and activated signaling pathways in developing cerebral cortex. Nat Biotechnol. 2014;32: 1053–1058.](http://paperpile.com/b/YTPw0C/NMHuU)

29. [Deng Q, Ramsköld D, Reinius B, Sandberg R. Single-cell RNA-seq reveals dynamic, random monoallelic gene expression in mammalian cells. Science. 2014;343: 193–196.](http://paperpile.com/b/YTPw0C/XZhcK)

30. [Kolodziejczyk AA, Kim JK, Tsang JCH, Ilicic T, Henriksson J, Natarajan KN, et al. Single Cell RNA-Sequencing of Pluripotent States Unlocks Modular Transcriptional Variation. Cell Stem Cell. 2015;17: 471–485.](http://paperpile.com/b/YTPw0C/Tiea7)

31. [Zeisel A, Muñoz-Manchado AB, Codeluppi S, Lönnerberg P, La Manno G, Juréus A, et al. Brain structure. Cell types in the mouse cortex and hippocampus revealed by single-cell RNA-seq. Science. 2015;347: 1138–1142.](http://paperpile.com/b/YTPw0C/o2xF)

32. [Zappia L, Phipson B, Oshlack A. Exploring the single-cell RNA-seq analysis landscape with the scRNA-tools database. PLoS Comput Biol. 2018;14: e1006245.](http://paperpile.com/b/YTPw0C/DAsA)
