# Supplementary figures and images for: Scedar: A scalable Python package for single-cell RNA-seq exploratory data analysis
Source: PLoS Comput Biol. 2020 Apr 27;16(4):e1007794. doi: 10.1371/journal.pcbi.1007794 (PMC7217489; doi:10.1371/journal.pcbi.1007794)

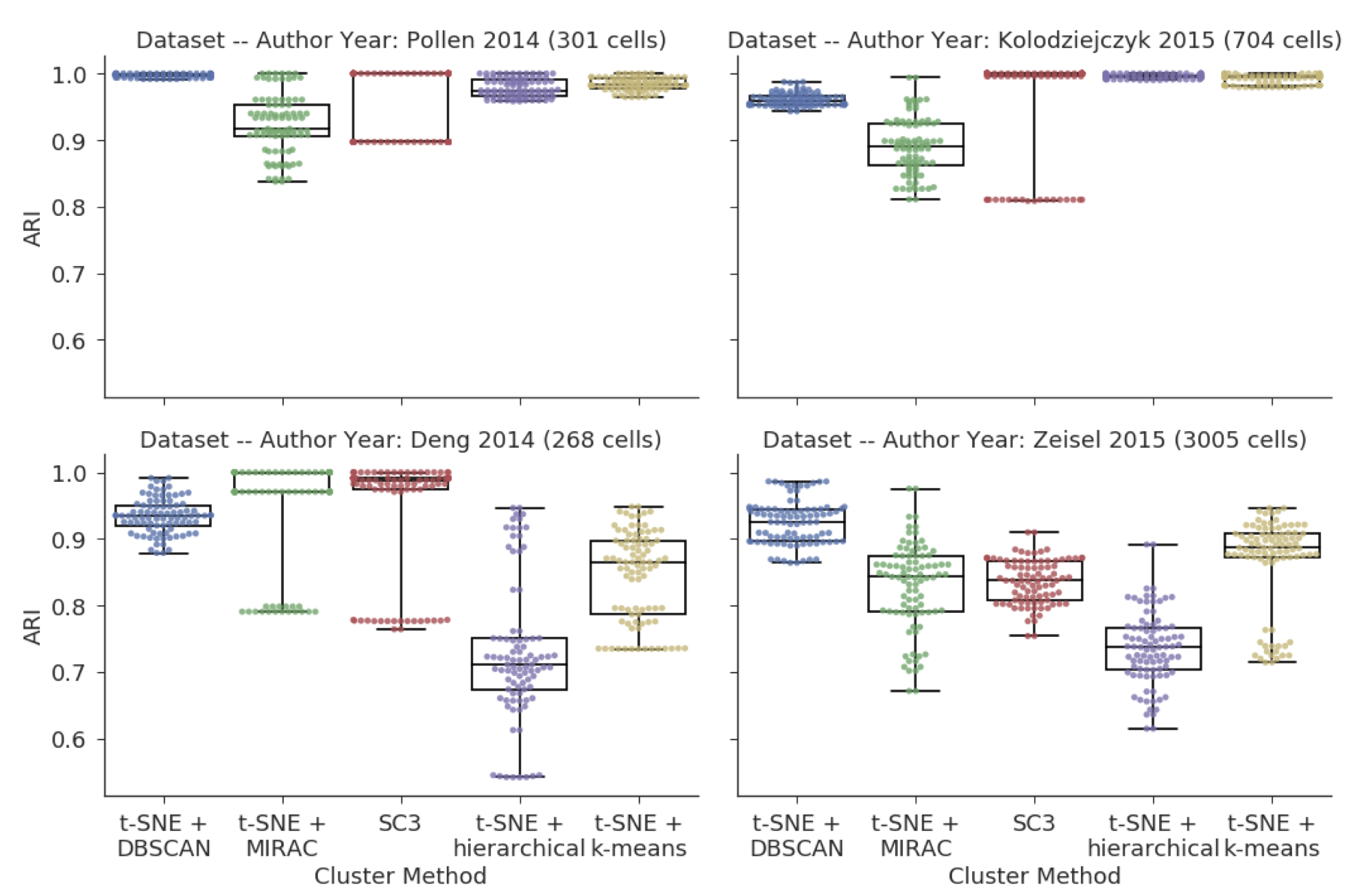

Supplement: S1 Fig — Similarity between clustering results generated with different random states but the same parameters, quantified by adjusted rand index (ARI) [23]. (TIF) [file pcbi.1007794.s002.tif]

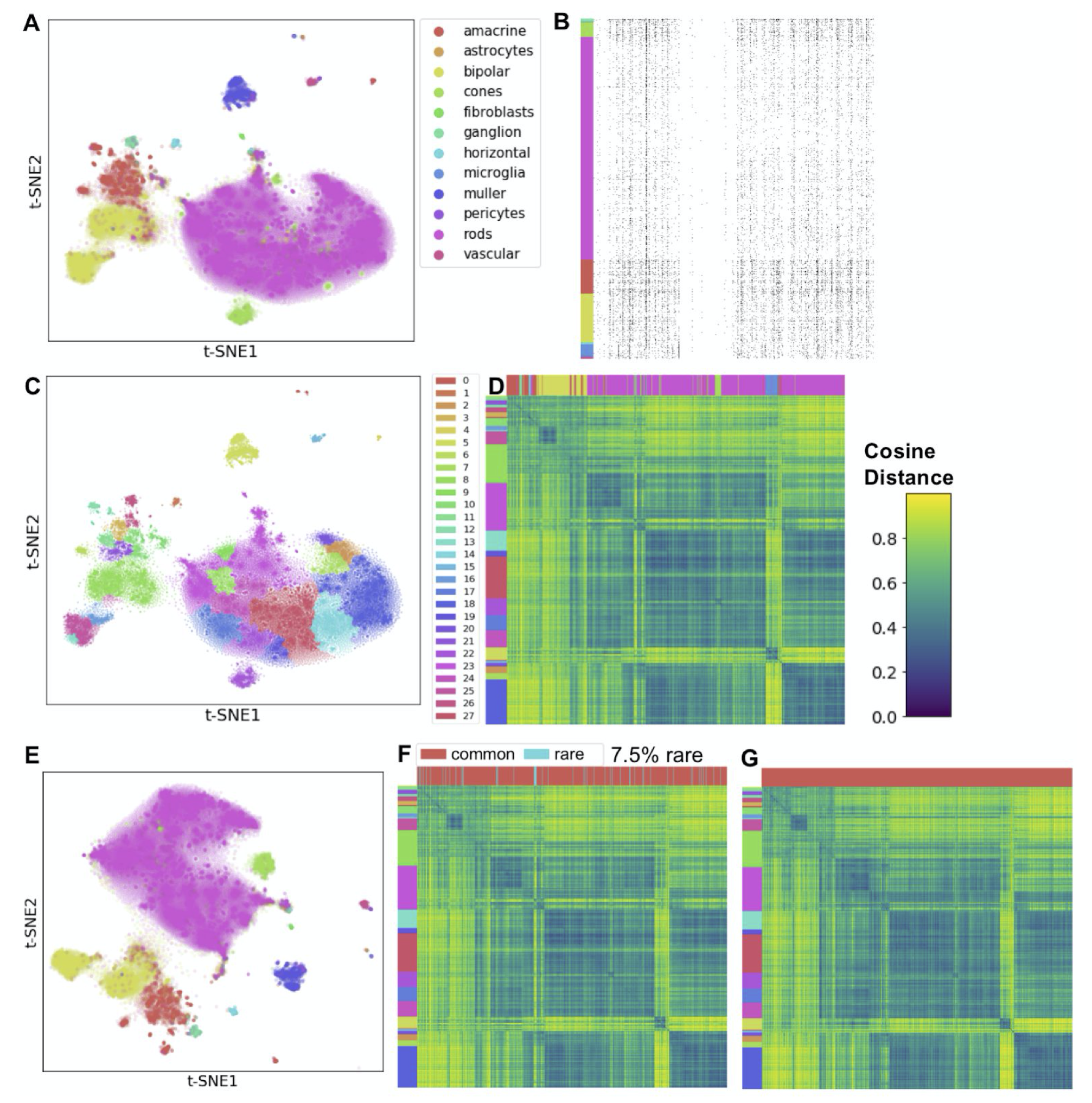

Supplement: S2 Fig — (A) t-SNE scatter plot with cell type labels. (B) Read count matrix heatmap with rows as cells, columns as genes, and black color as ≥ 1reads. (C) t-SNE scatter plot with MIRAC labels. (D) Pairwise cosine distance heatmap with left strip as MIRAC labels and upper strip as cell type labels. (E) t-SNE scatter plot after KNN gene dropout imputation with cell type labels. (F) pairwise cosine distance heatmap with left strip as MIRAC labels and upper strip as common or rare transcriptomic profile labels. (G) pairwise cosine distance heatmap with rare transcriptomic profiles removed. (TIF) [file pcbi.1007794.s003.tif]

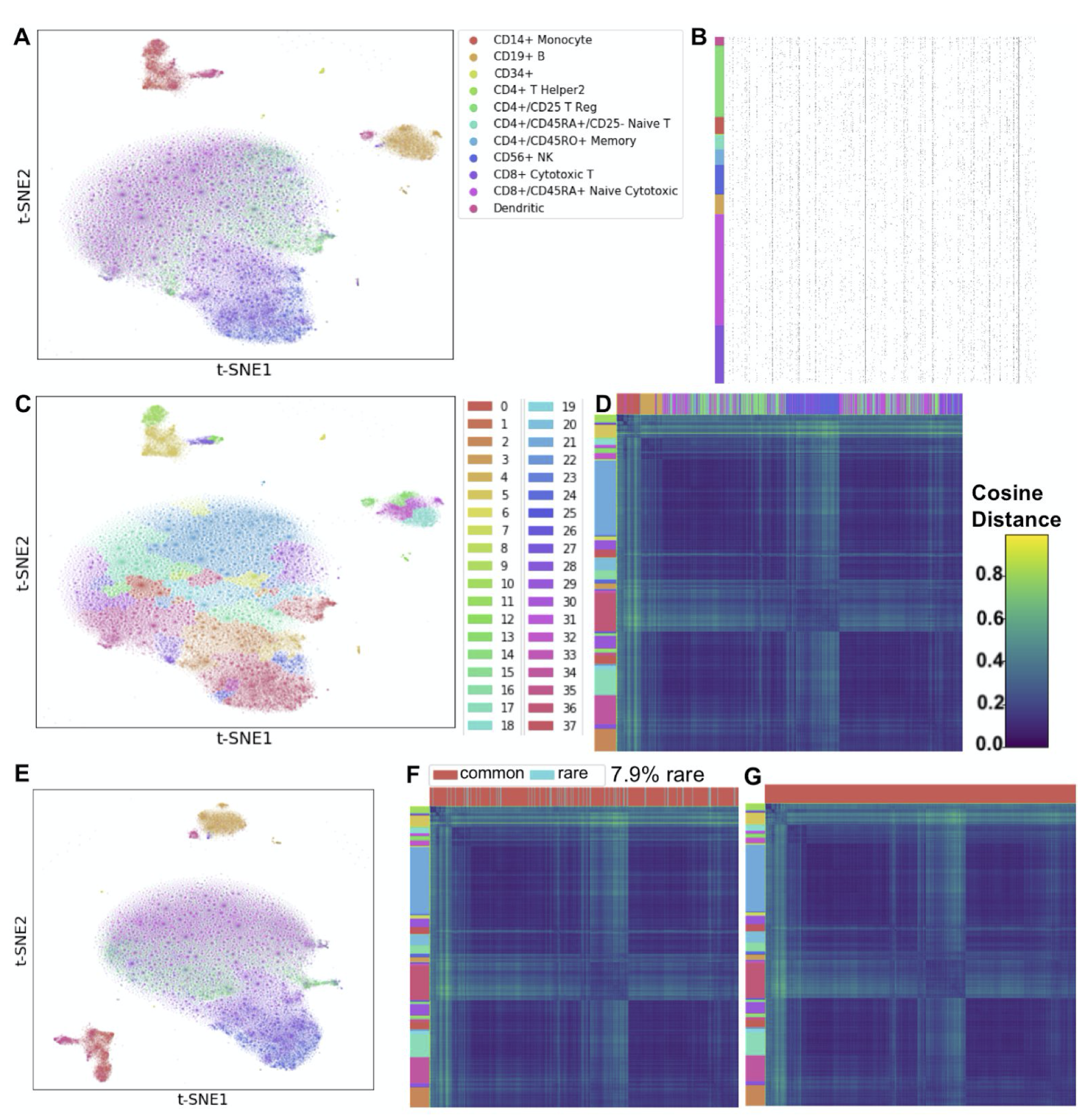

Supplement: S3 Fig — (A) t-SNE scatter plot with cell type labels. (B) Read count matrix heatmap with rows as cells, columns as genes, and black color as ≥ 1 reads. (C) t-SNE scatter plot with MIRAC labels. (D) Pairwise cosine distance heatmap with left strip as MIRAC labels and upper strip as cell type labels. (E) t-SNE scatter plot after KNN gene dropout imputation with cell type labels. (F) Pairwise cosine distance heatmap with left strip as MIRAC labels and upper strip as common or rare transcriptomic profile labels. (G) Pairwise cosine distance heatmap with rare transcriptomic profiles removed. (TIF) [file pcbi.1007794.s004.tif]

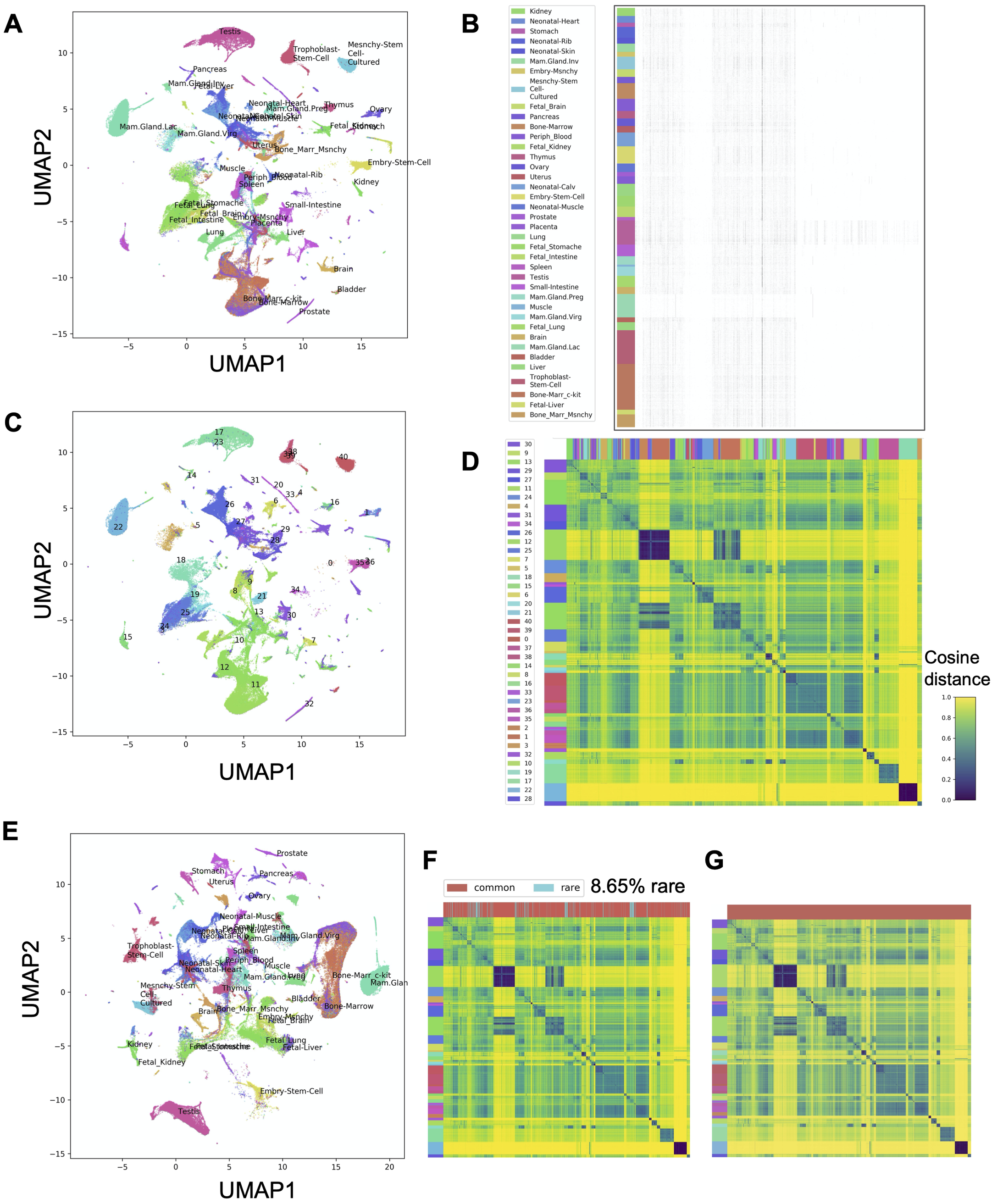

Supplement: S4 Fig — (A) UMAP scatter plot with cell type labels. (B) Read count matrix heatmap with rows as cells, columns as genes, and black color as ≥ 1 reads. (C) UMAP scatter plot with MIRAC labels. (D) Pairwise cosine distance heatmap of downsampled 7449 cells with left strip as MIRAC labels and upper strip as cell type labels. (E) UMAP scatter plot after KNN gene dropout imputation with cell type labels. (F) Pairwise cosine distance heatmap of downsampled 7449 cells with left strip as MIRAC labels and upper strip as common or rare transcriptomic profile labels. (G) Pairwise cosine distance heatmap of downsampled 7449 cells with rare transcriptomic profiles removed. (TIF) [file pcbi.1007794.s005.tif]

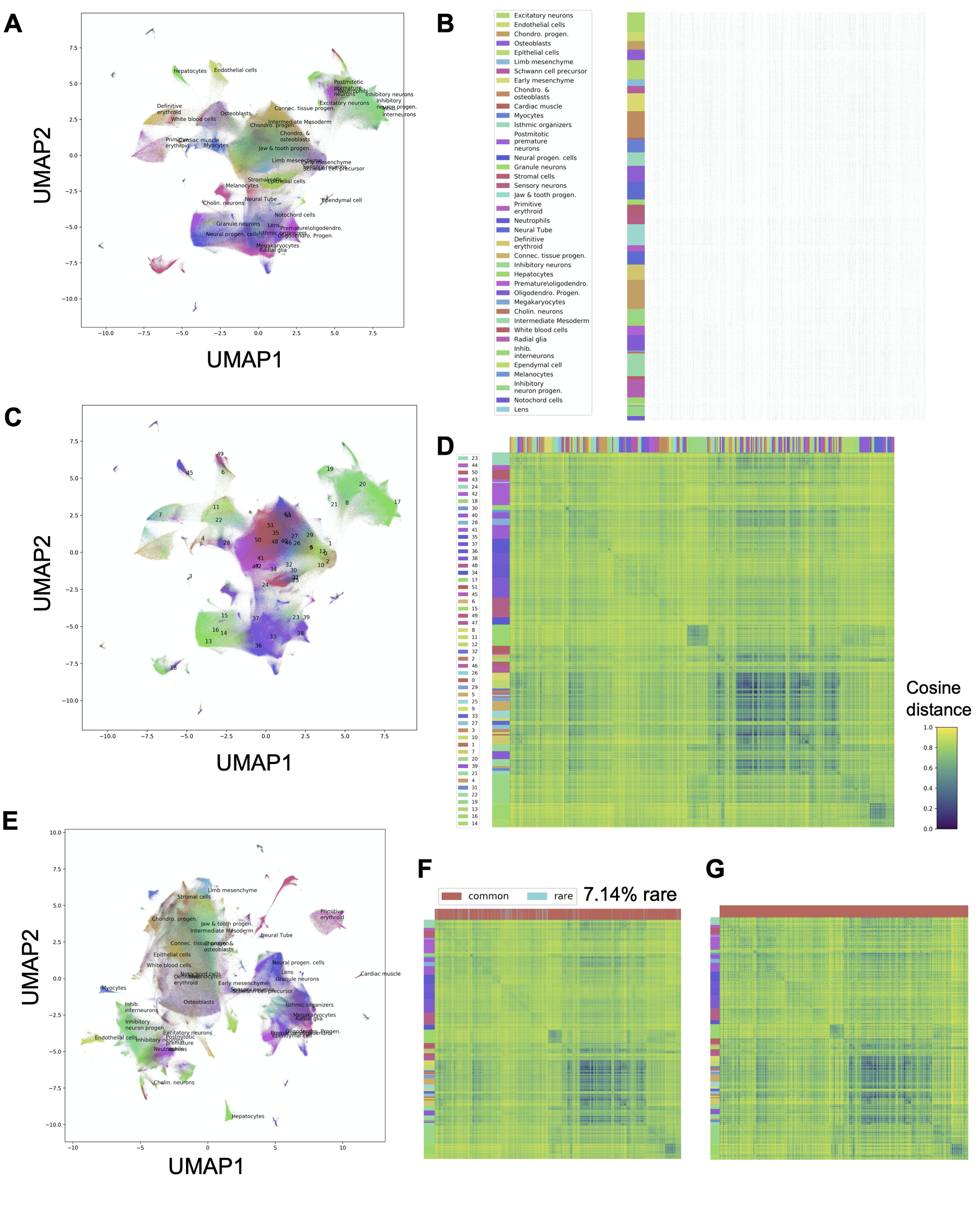

Supplement: S5 Fig — (A) UMAP scatter plot with cell type labels. (B) Read count matrix heatmap of downsampled 20,403 cells with rows as cells, columns as genes, and black color as ≥ 1 reads. (C) UMAP scatter plot with MIRAC labels. (D) Pairwise cosine distance heatmap of downsampled 20,118 cells with left strip as MIRAC labels and upper strip as cell type labels. (E) UMAP scatter plot after KNN gene dropout imputation with cell type labels. (F) Pairwise cosine distance heatmap of downsampled 20,118 cells with left strip as MIRAC labels and upper strip as common or rare transcriptomic profile labels. (G) Pairwise cosine distance heatmap of downsampled 20,118 cells with rare transcriptomic profiles removed. (TIF) [file pcbi.1007794.s006.tif]

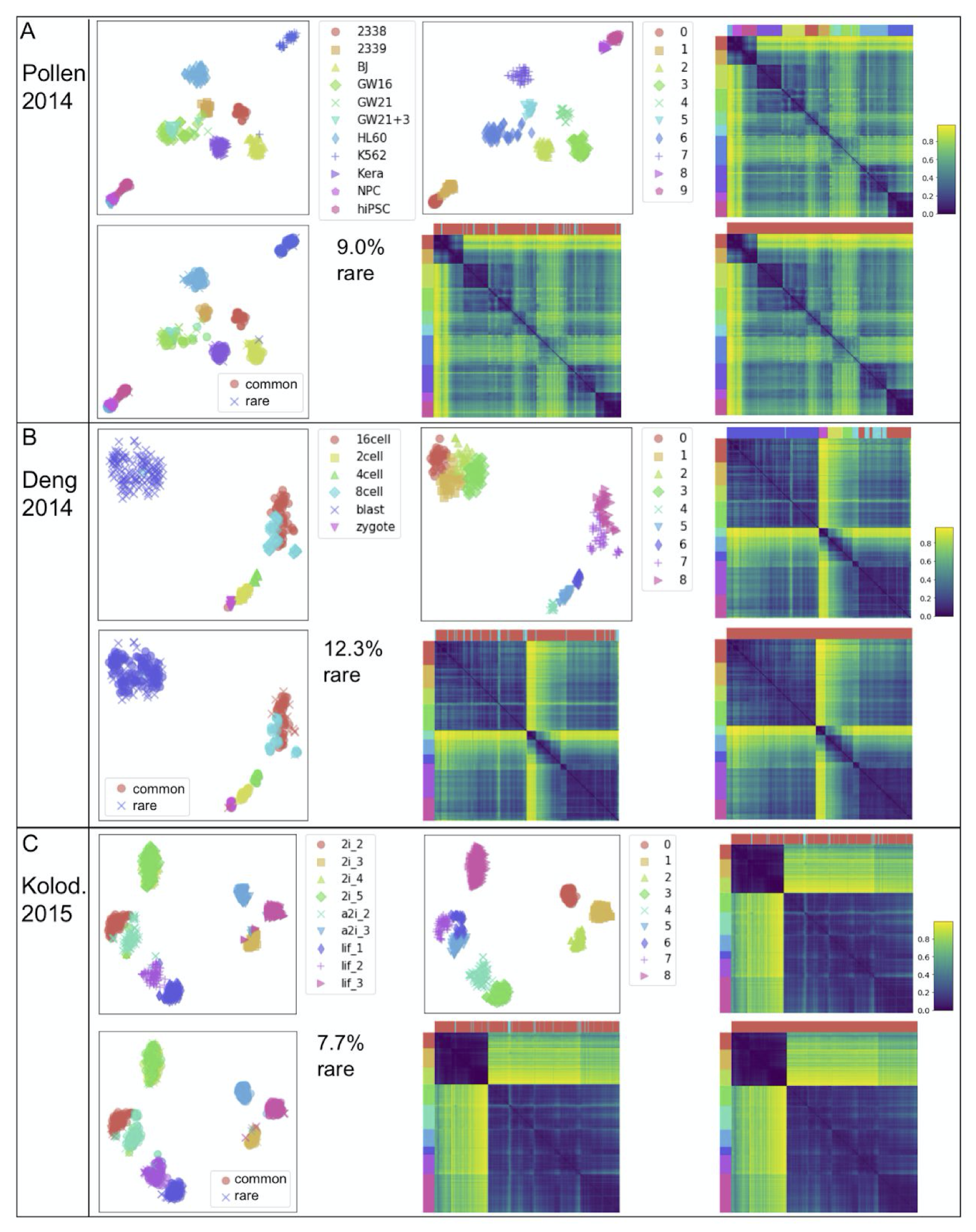

Supplement: S6 Fig — The sub-figures A, B, and C represent the results of scRNA-seq datasets published by Pollen et al. [28], Deng et al. [29], and Kolodziejczyk et al. [30] respectively. Within each sub-figure, the plots are 1) t-SNE scatter plot with cell type labels, 2) t-SNE scatter plot with MIRAC cluster labels, 3) pairwise cosine distance heatmap with left strip as MIRAC labels and upper strip as cell type labels, 4) t-SNE scatter plot with common or rare rare transcriptomic profile labels, 5) pairwise cosine distance heatmap with left strip as MIRAC labels and upper strip as common or rare transcriptomic profile labels, 6) pairwise cosine distance heatmap with rare transcriptomic profiles removed. (TIF) [file pcbi.1007794.s007.tif]

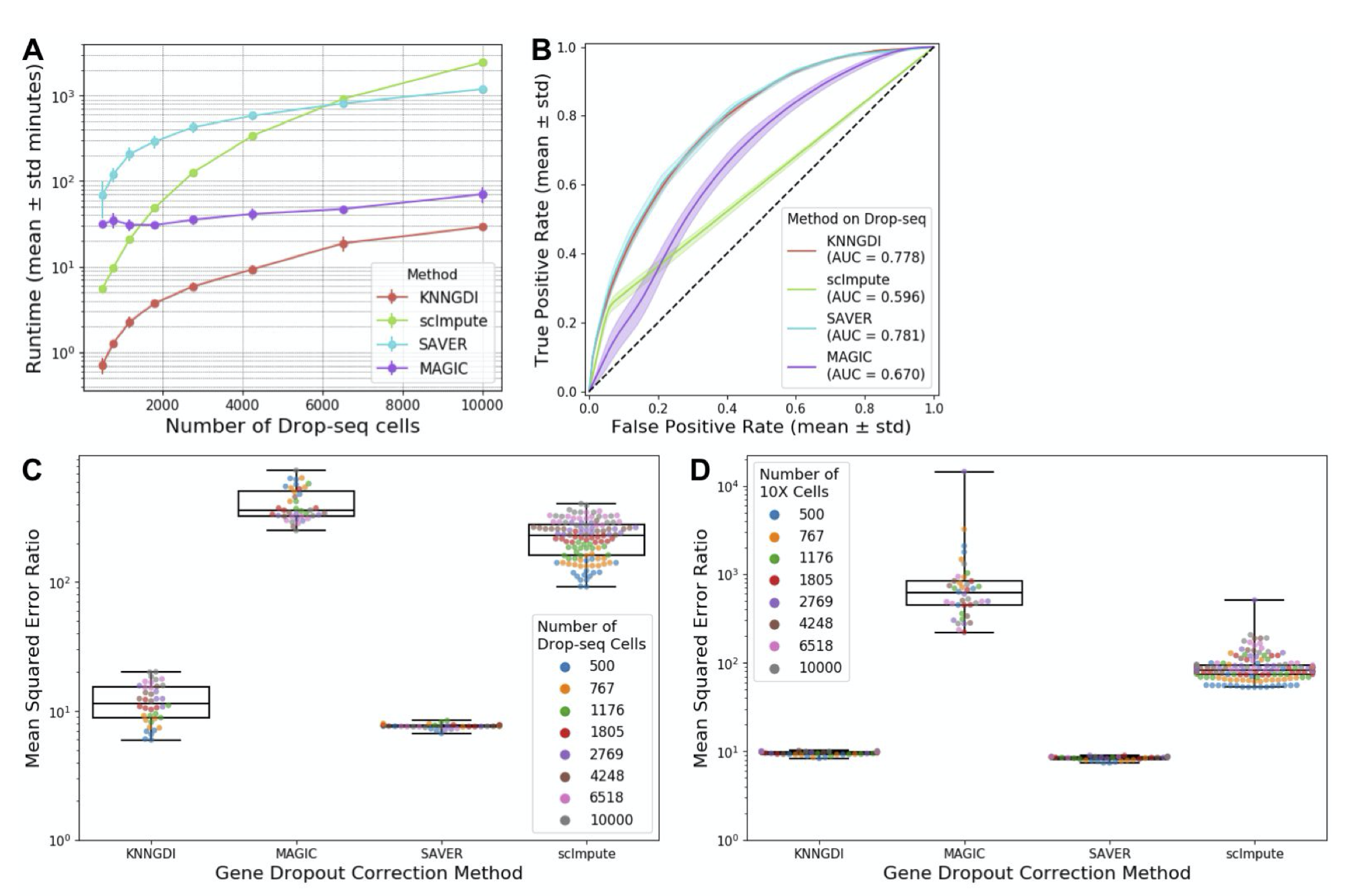

Supplement: S7 Fig — (A) Runtimes on 40 simulated Drop-seq datasets. (B) ROC curves (± standard deviation) of dropout detection on simulated Drop-seq datasets. (C) and (D) are mean squared error (MSE) ratios of different methods on simulated Drop-seq and 10x Genomics datasets respectively, where the MSE ratio is computed as the MSE of corrected read counts / MSE of true read counts. (TIF) [file pcbi.1007794.s008.tif]

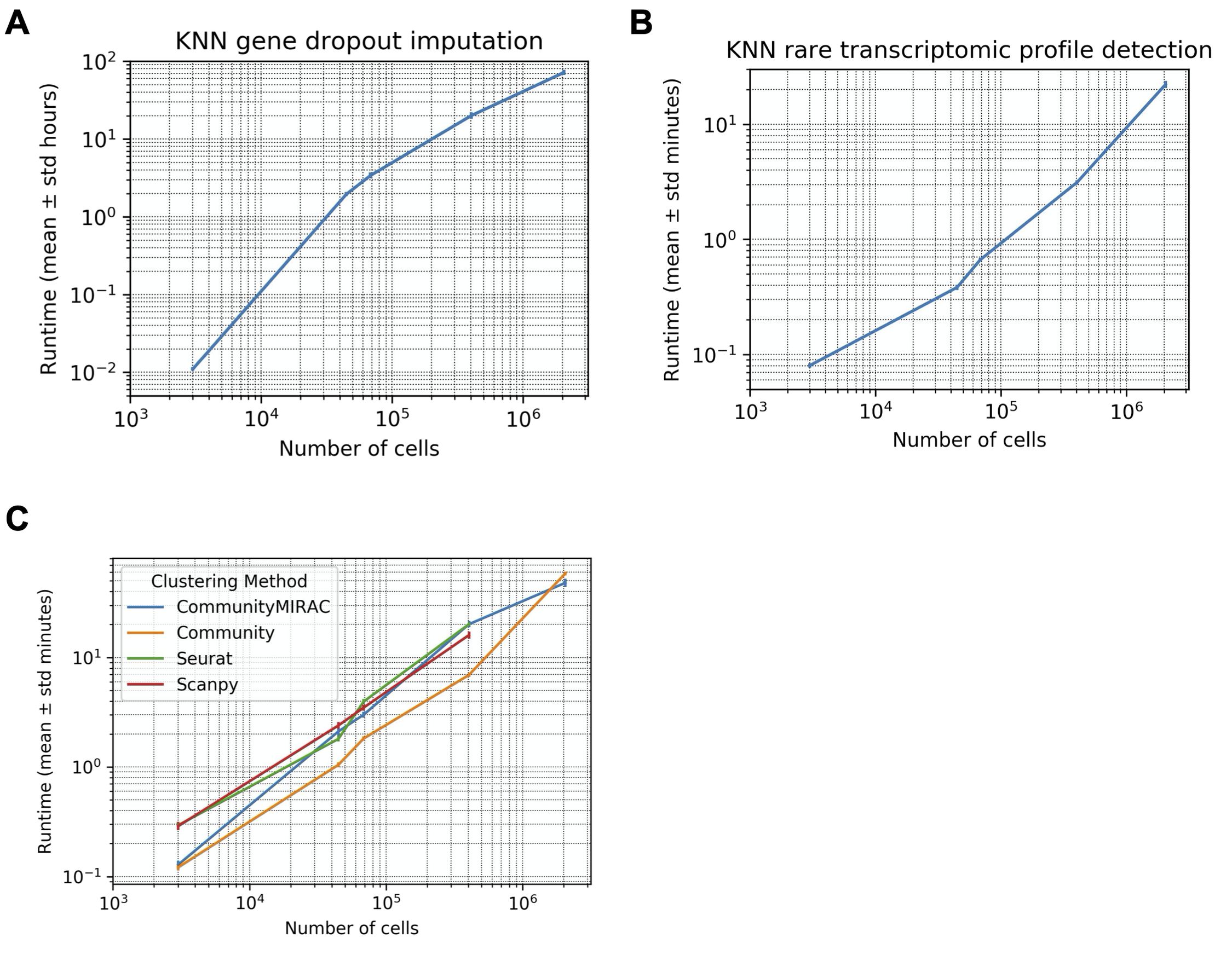

Supplement: S8 Fig — (A) KNN gene dropout imputation. (B) KNN rare transcriptomic profile detection. (C) Community clustering, community extended MIRAC clustering, Seurat clustering, and Scanpy clustering. These methods were all performed on experimentally generated scRNA-seq datasets with 3005, 44808, 68579, 405191, and 2058652 cells [24–27,31], except that Scanpy and Seurat were not able to cluster the mouse organogenesis cell atlas (MOCA) dataset that contain 2,058,652 single cells on a server with 1TB memory due to a mandatory conversion of the sparse read count matrix into dense matrix. (TIF) [file pcbi.1007794.s009.tif]

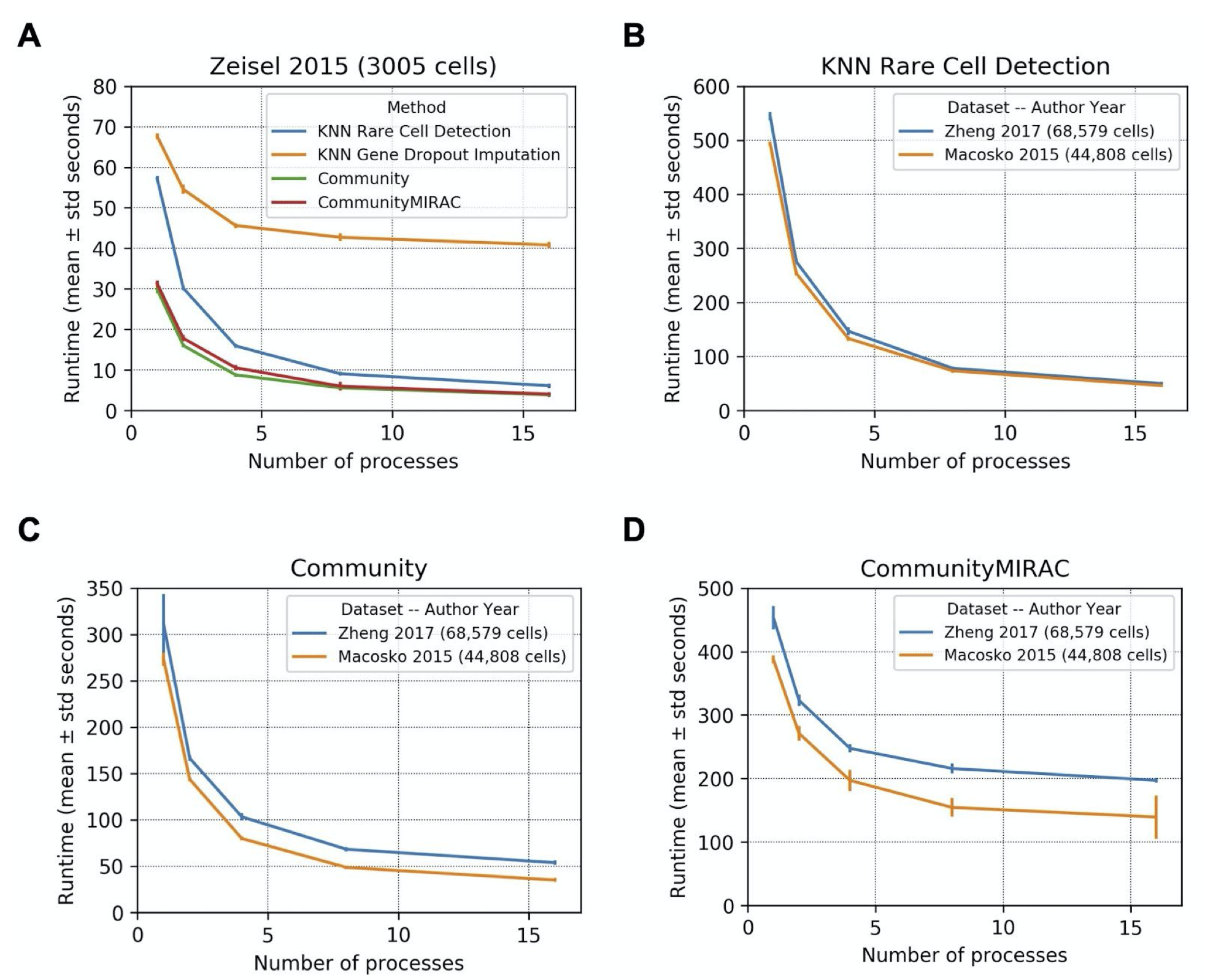

Supplement: S9 Fig — (A) All implemented methods performed on an scRNA-seq dataset with 3005 single cells [31]. (B) KNN rare transcriptomic profile detection, (C) community clustering, and (D) community extended MIRAC clustering performed on two scRNA-seq datasets with 44,808 and 68,579 single cells [24,25]. (TIF) [file pcbi.1007794.s010.tif]

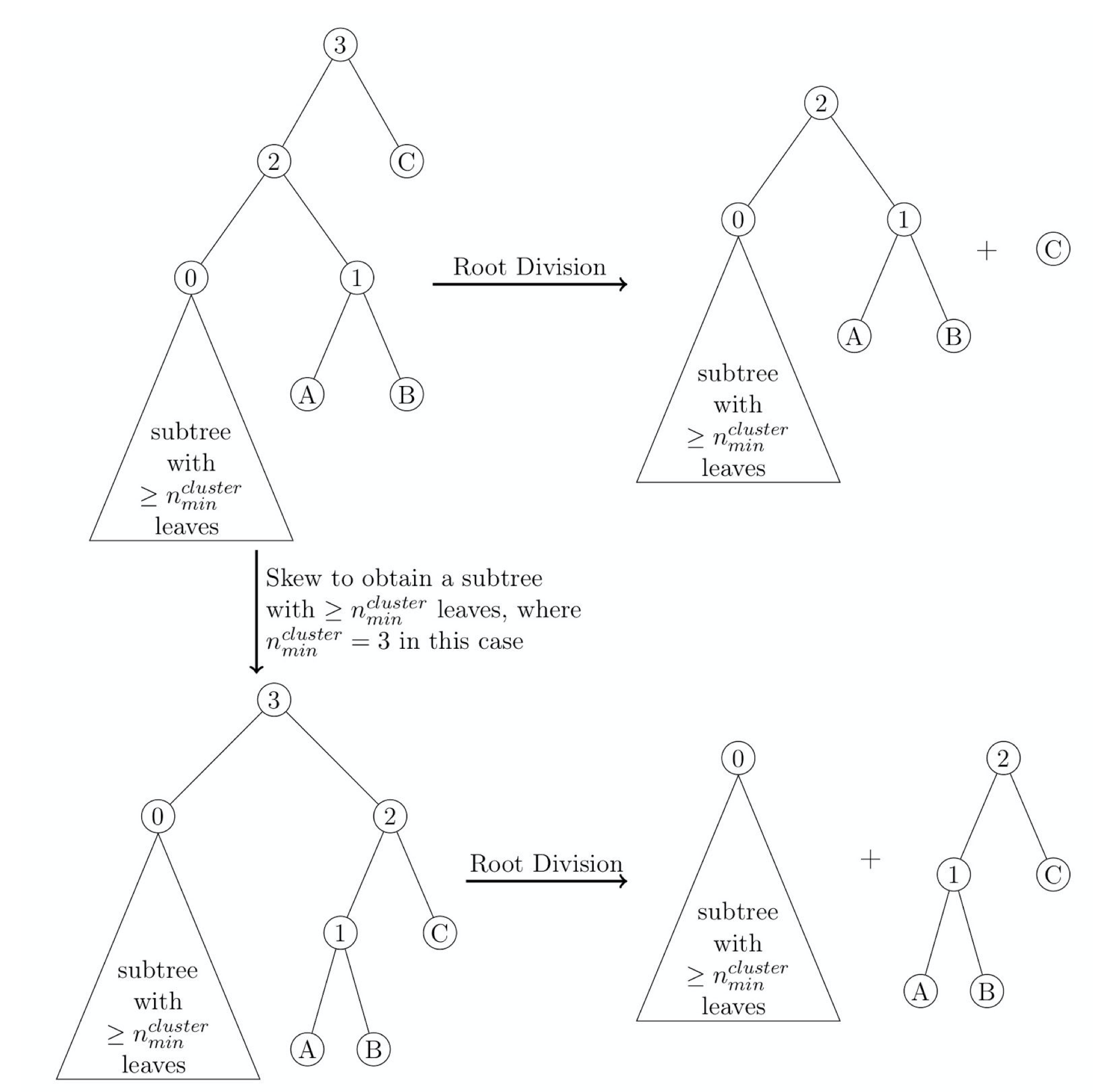

Supplement: S10 Fig — Tree leaves are samples, which are marked by upper case letters. Tree inner nodes are agglomerated samples by arbitrary linkage, which are marked by number. The triangle under inner node 0 represents an arbitrary valid subtree with ≥nmincluster leaves. The root division procedure divides a tree into left and right subtrees of the root node. The skewing procedure creates a minimum subtree of the root with ≥nmincluster leaves, where nmincluster=3 in this specific case. (TIF) [file pcbi.1007794.s011.tif]
